# Human Milk Feeding and Preterm Infants’ Growth and Body Composition: A Literature Review

**DOI:** 10.3390/nu12041155

**Published:** 2020-04-21

**Authors:** Jacopo Cerasani, Federica Ceroni, Valentina De Cosmi, Alessandra Mazzocchi, Daniela Morniroli, Paola Roggero, Fabio Mosca, Carlo Agostoni, Maria Lorella Giannì

**Affiliations:** 1 Department of Clinical Sciences and Community Health, University of Milan, 20122 Milan, Italy; jacopo.cerasani@unimi.it (J.C.); federica.ceroni@unimi.it (F.C.); valentina.decosmi@gmail.com (V.D.C.); paola.roggero@unimi.it (P.R.); fabio.mosca@unimi.it (F.M.); maria.gianni@unimi.it (M.L.G.); 2 Fondazione IRCCS Ca’ Granda Ospedale Maggiore Policlinico, Pediatric Intermediate Care Unit, 20122 Milan, Italy; alessandra.mazzocchi1@gmail.com; 3 Fondazione IRCCS Ca’ Granda Ospedale Maggiore Policlinico, NICU, 20122 Milan, Italy; daniela.morniroli@policlinico.mi.it

**Keywords:** human milk feeding, growth, body composition, preterm infant

## Abstract

Preterm infants may show a higher risk of adverse health outcomes, such as the development of metabolic syndrome and cognitive impairment. The most recent evidence highlights that nutrition, body composition development, and early postnatal growth may play a role in the programming of these processes. Human milk feeding has been recommended as the natural feeding for preterm infants and as a cost-effective strategy for reducing disease and economic burden. Considering that the postnatal growth retardation and aberrant body composition shown by preterm infants at the time of hospital discharge still remain important issues, we performed a literature review, aiming to provide an update about the effect of human milk feeding on these processes. On the basis of our findings, human milk feeding in preterm infants, although related to a slower weight gain than formula feeding, is associated with a better recovery of body composition through the promotion of fat-free mass deposition, which may ultimately lead to better metabolic and neurodevelopmental outcomes. Promotion and support of human milk feeding should be considered a priority in preterm infants’ care.

## 1. Introduction

Preterm birth represents a worldwide epidemic, accounting for an increased risk of mortality and morbidity both in the short and long term [1]. Innovative and effective strategies have been advocated to prevent preterm birth and to reduce its disease and economic burden [2]. Among the feasible and cost-effective proposed interventions, the promotion of human milk feeding has been strongly advocated due to its acknowledged health benefits in the preterm population [3,4]. However, the specific and complex mechanisms underlying the positive biological effects associated with human milk feeding have not been completely elucidated yet [5]. 

Preterm infants complete their organogenesis being exposed to non-physiological environmental triggers, including hospitalization in the neonatal intensive care unit and the occurrence of co-morbidities. As a consequence, their developing organism needs to adapt the metabolic and homeostatic pathways to the newly acquired extrauterine environment, resulting in an increased risk of developing an adverse metabolic and neurodevelopmental outcome later in life [6]. A huge amount of evidence indicates that early postnatal growth and body composition development may participate in the programming of these processes [7,8]. 

In view of the postnatal growth retardation and the aberrant body composition shown by preterm infants at the time of hospital discharge [7,9], a comprehensive knowledge of the modifiable determinants of these processes is fundamental to implement adequate strategies aimed at growth and body composition recovery. Given the strict interrelationship between the mode of feeding and growth [10], we performed a literature review aiming for providing an update about the effect of human milk feeding on growth and body composition development in preterm infants.

## 2. Materials and Methods

**Figure 1:**
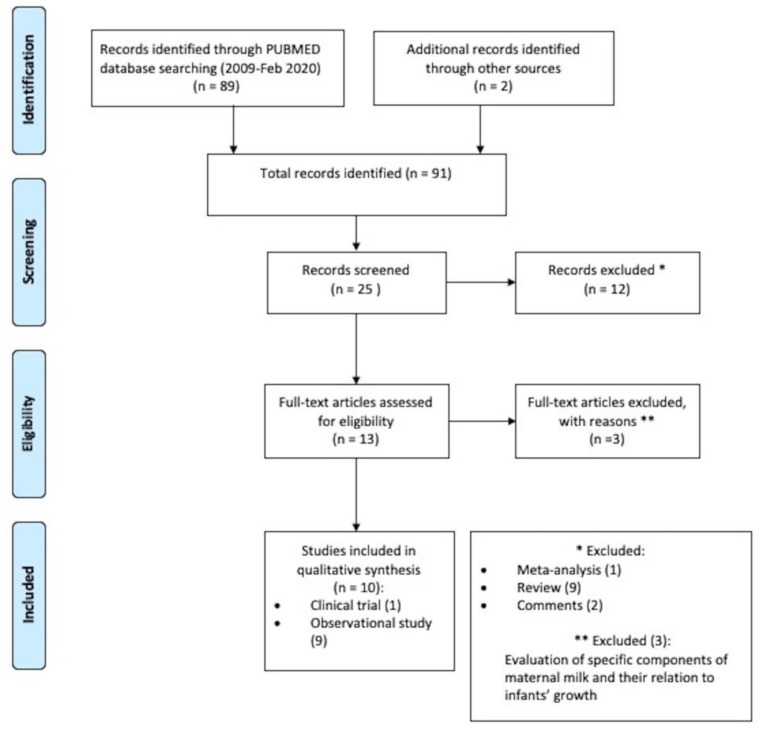
PRISMA 2009 research method flowchart.

The search strategy was conducted on 3 February 2020 using PUBMED, searching for trials published in English from 2009 onwards. The following key words were entered: (“human milk” OR “donor milk” OR “breast milk”) AND (“preterm infants” OR “premature infants” OR “very premature babies” OR “very preterm babies” OR “very low birth weight infants”) AND (“body composition” OR “body-mass index” OR “infant growth” OR “growth acceleration” OR “weight acceleration” OR “fat-free mass” OR “fat mass” OR “adipose tissue” OR “fatty tissue” OR “lean mass”). The outcome of the search led us to 89 articles. After reviewing the bibliography of these publications, two more studies were identified, thus counting up a final number of 91 articles. Among them, 25 articles were screened and 12 were excluded on the basis of the title and the abstract. Two researchers independently assessed for eligibility the retrieved 13 articles. To be eligible for inclusion, studies had to be either observational or clinical trials and to examine the effect of any amount of human milk, either the own mother’s or donor milk, on premature infants’ growth and/or body composition. Meta-analysis, reviews, and comments were excluded. If any disagreement occurred, an additional third researcher was involved in order to reach consensus. Finally, 10 studies were selected: One clinical trial and 9 observational studies. The selection procedures utilized for identifying and including the studies are shown in Figure 1.

## 3. Results

### 3.1. Human Milk Feeding and Growth Parameters

The growth of very low birth weight newborns was analysed by Colaizy et al. [11], who focused on the impact of increasing proportions of human milk and on the effects of a predominantly maternal milk versus predominantly donor human milk diets. The group of infants fed >75% human milk demonstrated a broader reduction in the weight z-score from birth to discharge compared to infants receiving < 75%. When analyzing the type of human milk (maternal, donor, or mixed maternal and donor milk), a trend emerged towards higher rates of growth-retarded infants at discharge in those receiving donor milk compared to those fed with >75% donor milk compared to those fed with either >75% maternal and maternal and donor milk. 

Verd et al. [12] assessed the suitability of an exclusive human milk diet, defined as mother’s milk supplemented with any amount of donor milk, versus formula feeding, defined as mother’s milk supplemented with any amount of formula, in extremely low birth weight infants before or after implementing a donor human milk protocol. They observed no significant differences in the weight, length, or head circumference z-score from birth to discharge for human milk versus formula-fed infants. 

The link between donor human milk, own mother’s milk, and preterm formula and the rate of growth was also analyzed by Brownell et al. [13]. Data showed that for every 10% rise of donor human milk intake, a reduction of the adjusted mean growth velocity for weight was detected. Furthermore, the adjusted mean change in the weight z-score decreased with increasing amounts of donor human milk but improved with an increasing proportion of formula intake. Finally, a negative correlation between donor milk intake and mean adjusted head circumference velocity was described. Table 1 reports the main findings of the studies included in the review that examined the effect of human milk feeding on the growth of preterm infants.

### 3.2. Human Milk Feeding and Body Composition Parameters

The impact of a human milk-based diet on the lean and fat mass in late preterm infants was studied by Giannì et al. [14]. They found a positive correlation between the consumption of human milk and adipose tissue mass deposition, which strengthens when reaching the term CGA. The study of Piemontese et al. [15] also investigated the link between the type of feeding and body composition in VLBW preterm infants. A higher percentage of lean mass deposition at term CGA was present in those VLBW infants fed with human milk at more than or equal to 50% of their total milk volume in comparison to those VLBW infants who were fed with human milk at less than 50%. More specifically, Morlacchi et al. [16] studied the protein contribution of the human milk to the body composition of the VLBW premature neonates. Despite being given comparable nutrient amounts, fortified human milk-fed infants demonstrated a higher nitrogen balance at discharge and a higher percentage of lean mass at term CGA when compared to the infants receiving formula milk. Furthermore, at multiple linear regressions, non-adipose tissue mass was independently associated with human milk feeding. Mól et al. [17] compared premature newborns fed with either their own mothers’ milk or formula to a control group of full-term newborns. They showed a reduced lean mass and a higher adipose mass in the group of VLBW preterm infants fed with formula milk only in comparison to the term infant group. On the other side, at term-corrected gestational age, the group of VLBW infants who were fed with maternal milk had an analogous body composition to the full-term newborns. Table 2 reports the main findings of the studies included in the review that examined the effect of human milk feeding on the body composition of preterm infants.

### 3.3. Human Milk Feeding and Growth and Body Composition Parameters

Growth and body composition were studied by Beliaeva et al. [18] who included and divided premature infants in three groups on the basis of different types of feeding. The authors reported that body weight, length, head and chest circumference at discharge were lower in exclusively maternal fed premature infants than those recorded in infants who received formula feeding. Similar differences were detected in premature infants exclusively receiving maternal milk compared to infants receiving mixed feeding (maternal and formula milk feeding). However, a positive correlation between physical growth and lean body mass of the maternal milk fed infants was observed. On the contrary, evaluation of body composition showed that formula-fed premature infants presented higher fat body mass than exclusively maternal milk fed infants. Visuthranukul et al. [19] compared post-discharge growth, fat mass and metabolic outcomes of appropriate for gestational age (AGA) versus small for gestational age (SGA) VLBW infants fed an exclusive human milk-based diet at 12–15 months (visit 1) and 22 months (visit 2) of corrected gestational age (CGA). The analysis showed that the SGA group gained equal weight to the AGA group in the interval between discharge and visit 1 and showed a significantly higher BMI z-score from visit 1 to visit 2, also when adjusted for GA and diet at discharge. They concluded that, at 2 years of age, SGA premature infants who exclusively received human milk demonstrated greater compensatory growth with no rise in adipose tissue mass when compared to AGA infants. On the contrary, in the randomized controlled trial of Li et al. [20], predominantly formula fed infants weighted more than the exclusively human milk fed group at term corrected age and their greater change in weight Z-score throughout the study was accompanied by a higher non-adipose tissue mass deposition. No association was found between formula feeding and greater adiposity at term age. Table 3 reports the main findings of the studies included in the review that examined the effect of human milk feeding on growth and body composition of preterm infants.

## 4. Discussion

This literature review describes the effects of human milk feeding on growth and body composition in premature infants. According to the majority of the included studies assessing growth from birth either to discharge or term-corrected age [11,13,18], human milk feeding appears to be associated with suboptimal growth, with an inverse relationship between growth and the amount of human milk consumed, which becomes more pronounced with the use of donor milk as supplementation of own mother’s milk. On the other hand, all the authors [14,15,16,17,18], except one [20], reported that human milk feeding in preterm infants is positively associated with fat-free mass deposition, thus contributing to a positive recovery of body composition in this population. Moreover, catch-up growth following hospital discharge appears not to be associated with an increased deposition of fat mass when preterms infants either born small for gestational age or adequate for gestational age are exclusively fed human milk [19].

However, caution must be taken when interpreting these results since the included studies assessed the addressed outcomes through either a prospective or retrospective study design in all cases but one, thus providing a limited level of evidence. Moreover, they encompassed a heterogeneous population of premature infants, including both very preterm, moderate, and late preterm infants, who are characterized by different nutritional requirements and may exhibit specific patterns of body composition development throughout the first months of corrected age. Finally, growth was assessed using different growth charts and body composition was investigated by different techniques. 

Colaizy et al. [11] conducted a retrospective study, assessing growth in terms of weight in a relatively large sample size of very preterm infants. The authors reported a lower weight gain during hospital stay in infants fed with an amount of human milk > 75% compared to infants receiving human milk < 75%. Moreover, when taking into account the type of human milk feeding, a percentage greater than 75% of donor milk tended to be associated with higher rates of growth-retarded infants at discharge compared to those fed with either maternal and mixed human milk. However, the authors did not include the assessment of either length or head circumference, which could provide additional information on the quality of growth itself. Brownell et al. [13] retrospectively assessed anthropometric parameters of very preterm infants with a birth weight less than 1800 g from birth to discharge and reported a negative association between weight and head circumference growth with the amount of donor milk consumed as supplementation of maternal milk. In line with these findings, Beliaeva et al. [18] reported a suboptimal growth during hospital stay, in terms of weight, length, and head circumference of exclusively human milk-fed infants in comparison to formula-fed ones. However, it has to be taken into account that, in this observational study, the authors assessed a relatively limited number of infants, with a gestational age ranging from 26 to 36 weeks, thus including very preterm, moderate, and late preterm infants, who could have different nutritional needs according to the grade of prematurity itself. 

The present findings underline the importance of strictly monitoring preterm infants’ growth, especially when they are fed maternal milk supplemented with donor milk. The limited growth found in human milk-fed preterm infants may be at least partially explained by the fact that fortification of human milk has been performed using a standard approach, which has been demonstrated to lead to potential undernutrition, particularly regarding protein [21]. It must also be considered that the nutritional components of donor milk are affected by the processing required to ensure its safety [22]. Furthermore, donor milk is typically mature milk collected from mothers who have delivered at term. As a consequence, its macronutrient content, with particular regard to proteins, is lower that that of preterm milk and does not meet the high preterm infants’ nutritional needs unless adequately fortified [22]. Taking into account these aspects and in view of the high cost of donor milk, it has therefore been questioned whether its use is cost-effective or not [23]. However, donor milk feeding has been associated with a decreased risk of developing necrotizing enterocolitis, bronchopulmonary dysplasia, and late-onset sepsis, and its use is reccomended when own mother’s milk is not available or not sufficient [24,25].

Contrary to these findings, Verd et al. [12] demonstrated no difference in growth parameters at hospital discharge in extremely preterm infants according to being either exclusively human milk fed or formula fed. These results may partially reflect an increased awareness of health care professionals of the importance of promoting human milk feeding in preterm infants and closely monitoring their growth since the authors have conducted a multi-center pre-post retrospective study, before and after the implementation of a donor human milk policy. Moreover, Visuthranukul et al. [18] reported acceptable growth through the first two years of life in both small for gestational age and adequate for gestational age preterm infants fed human milk during their hospital stay, suggesting that human milk feeding supports adequate growth after discharge from the neonatal intensive care unit. 

Given the strict interrelationship between growth and neurodevelopment [26,27], the acknowledged improved cognitive outcome in preterm infants fed human milk in spite of the suboptimal weight gain reported by several authors has been referred to as the ‘apparent breastfeeding paradox’ [28]. When considering the association between human milk feeding and body composition development, however, most of the studies included in the present review reported an increased fat-free mass deposition in preterm infants fed human milk in comparison with formula-fed infants, suggesting that the promotion of fat-free mass deposition in preterm infants may represent one of the mechanisms involved in the protective effect exerted by human milk feeding towards obesity and adverse neurodevelopment outcomes. Accordingly, Mol et al. [17] conducted a prospective cohort study, assessing body composition in a limited sample of very preterm infants at term-corrected age, according to the mode of feeding during their hospital stay. The authors reported that formula-fed preterm infants showed lower fat-free mass deposition compared to full-term infants. On the contrary, the body composition of infants fed their own mother’s milk fortified with a standard approach was similar to that of full-term infants at birth. Piemontese et al. [15] reported that the effect of being fed human milk, either own mother’s or donor, on body composition appears to be dose dependent. The authors found that being fed human milk >50% of the total enteral intake was independently associated with fat-free mass deposition in very low birth weight infants assessed at term-corrected age. Remarkably, the authors performed human milk fortification following a targeted approach, which has been demonstrated to provide appropriate energy and protein intakes since it takes into account the variability of human milk macronutrient composition [21]. However, it has to be noted that the number of enrolled subjects was relatively limited and no separate analysis was conducted considering the percentage of either own mother’s milk or donor milk. The same research group further reported a dose-dependent effect of own mother’s milk feeding on fat-free mass deposition in late preterm infants [14]. This association became progressively stronger with the increased duration and amount of preterm infants’ exposure to own mother’s milk feeding [14]. In line with these findings, Morlacchi et al. [16] demonstrated a higher nitrogen balance and greater protein storage in a limited number of preterm infants fed with fortified own mother’s milk compared with formula-fed ones. These results indicate that, once adequate energy is provided, own mother’s milk protein can be utilized for anabolic purposes [10] as suggested by the direct relationship between own mother’s milk feeding and preterm infants’ fat-free mass content evaluated at term-corrected age [16]. On the contrary, Li et al. [20] conducted a secondary analysis of a randomized controlled trial and reported a higher weight and fat-free mass deposition in formula-fed preterm infants than in human milk-fed ones whereas no difference in fat mass content was found according to the mode of feeding. These results may be partially due to the fact that fortification was performed by the attending clinician without following a specific protocol, thus leading to the provision of protein and energy intakes that were inadequate to allow for fat-free mass deposition. Moreover, maternal milk and donor milk were pooled together for analysis.

Different studies agree that preterm birth per se poses a challenge on glucose and lipidic metabolism, especially for those infants who experience an accelerated weight gain [29,30,31]. Furthermore, the altered body composition shown by preterm infants at term-corrected age may contribute to the programming process, representing a risk factor for developing metabolic syndrome in early adulthood [7,8]. Body composition development further impacts on cognitive outcome as highlighted by the positive association found between fat-free mass z-scores at hospital discharge and Bayley Scales of Infant Development-III at 12 and 24 months of age [32]. 

Additional studies with larger cohorts, longer follow-up periods, and consistent evaluation methods are needed to gain further insight into the contribution of adequately fortified human milk in the modulation of preterm infants’ early growth, body composition, and related long-term outocomes.

## 5. Conclusions

On the basis of the present findings, we can therefore conclude that human milk feeding in preterm infants, although related to a slower weight gain than formula feeding, is associated with a better recovery of body composition through the promotion of fat-free mass deposition, which may ultimately lead to improved metabolic and neurodevelopmental outcomes. The promotion and support of human milk feeding should be considered a priority in preterm infants’ care.

## Figures and Tables

**Table 1:** Summary of the included studies about the effect of human milk on growth.

Study	Type of Study	Population	Timing of Evaluation	Principal Aim	Method of Assessment	Main Findings
Colaizy et al. [11]	Retrospective cohort study	*n* = 171 infants, VLBW ≤ 1250 g	From birth to discharge	Growth in VLBW fed with increasing proportions of HM Growth in VLBW infants predominantly MM vs. predominantly DM fed	Anthropometric measurements (z-scores calculated by Fenton growth charts)	Wider reduction in weight z-score from birth to discharge in infants fed with HM > 75% compared to infants receiving HM < 75% (−0.6 vs. −0.4, *p* = 0.03) Decrease in weight z-score from birth to discharge (*p* < 0.0001) in all the different groups of HM % intake
Verd et al. [12]	Multi-center pre-post retrospective study before and after implementation of a donor human milk policy	*n* = 201, EBLW < 1000 g	From birth to discharge	Growth in VLBW infants exclusively HM vs. FM fed	Anthropometric measurements (z-scores calculated by Fenton growth charts)	No difference of weight, length or HC z-score from birth to discharge between exclusively HM and FM-fed infants
Brownell et al. [13]	Single-centre retrospective study	*n* = 314, GA < 32 weeks or ≤ 1800 g	At 36 weeks or at hospital discharge	Growth in preterm infants receiving exclusively HM or any FM	Anthropometric measurements (z-scores calculated by revised Fenton growth charts)	Reduction of adjusted mean growth velocity for weight for every 10% rise of DM amount (β −0.17, 95% CI −0.28 −0.05, *p* = 0.01) Adjusted mean change in weight z score decreased with increasing amounts of DM (β −0.04 95% CI −0.06 −0.02, *p* < 0.001), but improved with increasing proportion of FM intake (β 0.03, 95% CI 0.01 0.05, *p* = 0.01) Reduction of mean adjusted HC velocity for every 10% rise of DM amount (β −0.01, 95% CI −0.02 −0.001, *p* = 0.03)

Human milk (HM) = own mother’s or maternal milk (MM) and donor milk (DM) fed; Formula milk = FM; Gestational age (GA); Very low birth weight infants (VLBW); Extremely low birth weight (ELBW); HC (head circumference); CI (Confidence Interval).

**Table 2:** Summary of the included studies about the effect of human milk on body composition.

Study	Type of Study	Population	Timing of Evaluation	Principal Aim	Method of Assessment	Main Findings
Giannì et al. [14]	Observational cohort study	*n* = 284, GA 34–36 weeks	At term CGA	Body composition in late preterms infants fed with exclusively MM/any MM vs. FM	Air-displacement pletysmograph (PEA POD Infant Body Composition System)	Positive association of any MM feeding at discharge and at term CGA and exclusively MM feeding at term CGA with FFM content (β =−47.9, 95%, CI = −95.7; −0.18; *p* = 0.049; β = −89.6, 95% CI = −131.5; −47.7; *p* < 0.0001; β= −104.1, 95% CI = −151.4; −56.7, *p* < 0.0001)
Piemontese et al. [15]	Longitudinal observational study	*n* = 73, VLBW< 1500 g, GA 26–34 weeks	At term CGA	Body composition in VLBW preterm infants fed with HM at < 50% vs. HM ≥ 50% of the total volume intake	PEA POD Infant Body Composition System	Positive association between the HM % and FFM % after correction for birth weight and gender (β = 0.12 ± 0.05, *p* = 0.01) Increase in FFM % at term GCA when HM ≥ 50% (*p* = 0.01)
Morlacchi et al. [16]	Prospective observational study	*n* = 32, VLBW< 1500 g, GA ≤ 32 weeks	At discharge and at term CGA	Body composition and protein balance in VLBW premature neonates exclusively MM vs. FM fed	PEA POD Infant Body Composition System Standard nitrogen balance method; Infrared spectroscopy analysis to assess nutritional composition of the MM; for FM, macronutrients calculated based on manufactures’ info	At discharge, higher nitrogen balance in MM-fed infants compared with FM fed (mean 488.3 ± 75 compared with 409.8 ± 85 mg kg^−1^ d^−1^, *p* = 0.009) At term CGA, in MM-fed compared to FM-fed infants higher FFM % (85.1 ± 2.8 vs. 80.8 ± 3.2, *p* = 0.002), lower ATM % (14.9 ± 2.8 vs. 19.2 ± 3.2, *p* = 0.002), lower ATM (458 ± 118, *p* = 0.004) FFM independently associated with MM feeding (*R^2^* = 0.93, *p* < 0.0001)
Mól et al. [17]	Prospective cohort study	*n* = 53, VLBW 1000–1500 g	At birth and at term CGA	Body composition of VLBW newborns fed with either MM or FM compared to full-term infants	Multi-frequency impedance body composition monitor	In the FM-fed VLBW preterms compared to full-term newborns lower FFM % (83.5 vs. 85.5, *p* < 0.001), higher ATM % (16.4 vs.14.5, *p* < 0.01) and higher ATM kg (0.617 ± 0.18 vs. 0.494 ± 0.1, *p* = 0.02) No differences in FFM or FM between the HM fed VLBW infants and the term newborns

Human milk (HM) = own mother’s or maternal milk (MM) and donor milk (DM) fed; Formula milk = FM; Gestational age (GA); Corrected gestational age (CGA); Very low birth weight infants (VLBW); Adipose tissue mass (ATM); Fat-free mass (FFM); CI (Confidence Interval).

**Table 3:** Summary of the included studies about the effect of human milk on growth and body composition.

Study	Type of Study	Population	Timing of Evaluation	Principal Aim	Method of Assessment	Main Findings-Growth	Main Findings Body-Composition
Beliaeva et al. [18]	Observational cohort study	*n* = 80, GA 28–36 weeks	From birth to discharge	Growth and body composition in premature infants fed with FM vs. FM + MM vs. exclusively MM	Anthropometric measuraments (z-score calculated by ANTHRO-WHO 2009) Air-displacement pletysmography (PEA POD Infant Body Composition System)	Lower body weight, length, head and chest circumference at discharge in exclusively MM-fed infants compared to infants receiving FM (*p* < 0.05) Similar differences in premature infants (GA < 34 weeks) receiving exclusively MM compared to MM + FM-fed infants (*p* < 0.05)	Higher ATM in FM group compared to MM fed infants (*p* < 0.05)
Visuthranukul et al. [19]	Single-centre longitudinal cohort study	*n* = 51, VLBW ≤ 1250 g, GA < 37 weeks	At 12–15 months (visit 1) and at 18–22 months CGA (visit 2)	Growth and body composition of VLBW SGA vs. AGA exclusively fed with HM	Anthropometric measurements with centile (WHO growth data) Dual-energy X-ray absorptiometry	SGA greater BMI z-score gain from visit 1 to visit 2 (0.25 ± 1.10 vs. −0.21 ± 0.84, *p* = 0.02), also after controlling for GA and diet at discharge (*p* = 0.004) In both SGA and AGA groups, from birth to visit 2: increase of weight (from 3rd-25th pcl to 25th pcl; from 25th pcl to 50th pcl, respectively, *p* = 0.022) and HC (from 3rd-10th pcl to 25th pcl; from 10th-25th pcl to 50th pcl, respectively, *p* = 0.002)	No difference in body composition between the two groups
Li et al. [20]	Preplanned secondary analysis of the Nutritional Evaluation and Optimisation in Neonates (NEON) trial	*n* = 133, GA < 31 weeks	At birth and at term CGA	Body composition at term CGA in very preterm infants HM vs. FM fed	Anthropometric measurements (growth charts used for calculation of z-scores not mentioned) Whole body MRI	Predominantly FM group weighed more than the exclusively HM-fed group, mean difference 283.6 g (95% CI, 121.6–445.6) Greater positive weight Z-score change between birth and term CGA in predominantly FM group compared to exclusively HM-fed group, mean difference 0.6 (95% CI, 0.2–1.0), *p* < 0.01	Higher FFM in predominantly FM-fed group than in exclusively HM-fed group, mean difference 257.4 g (95% CI, 139.1–375.7 g), *p* < 0.01 No significant differences between exclusively HM-fed group in ATM or % ATM and the predominantly HM and predominantly FM-fed groups, respectively.

Human milk (HM) = own mother’s or maternal milk (MM) and donor milk (DM) fed; Formula milk = FM; Gestational age (GA); Corrected gestational age (CGA); Small for gestational age (SGA); Appropriate for gestational age (AGA); Very low birth weight infants (VLBW); Adipose tissue mass (ATM); Fat-free mass (FFM); BMI (body mass index), HC (head circumference), CI (Confidence Interval).
